# Supplementary material for: 2.4 GHz Electromagnetic Field Influences the Response of the Circadian Oscillator in the Colorectal Cancer Cell Line DLD1 to miR-34a-Mediated Regulation
Source: Int J Mol Sci. 2022 Oct 30;23(21):13210. doi: 10.3390/ijms232113210 (PMC9656412; doi:10.3390/ijms232113210)
Supplement: Supplementary file 1 [file ijms-23-13210-s001.zip › Figure S1.pdf]

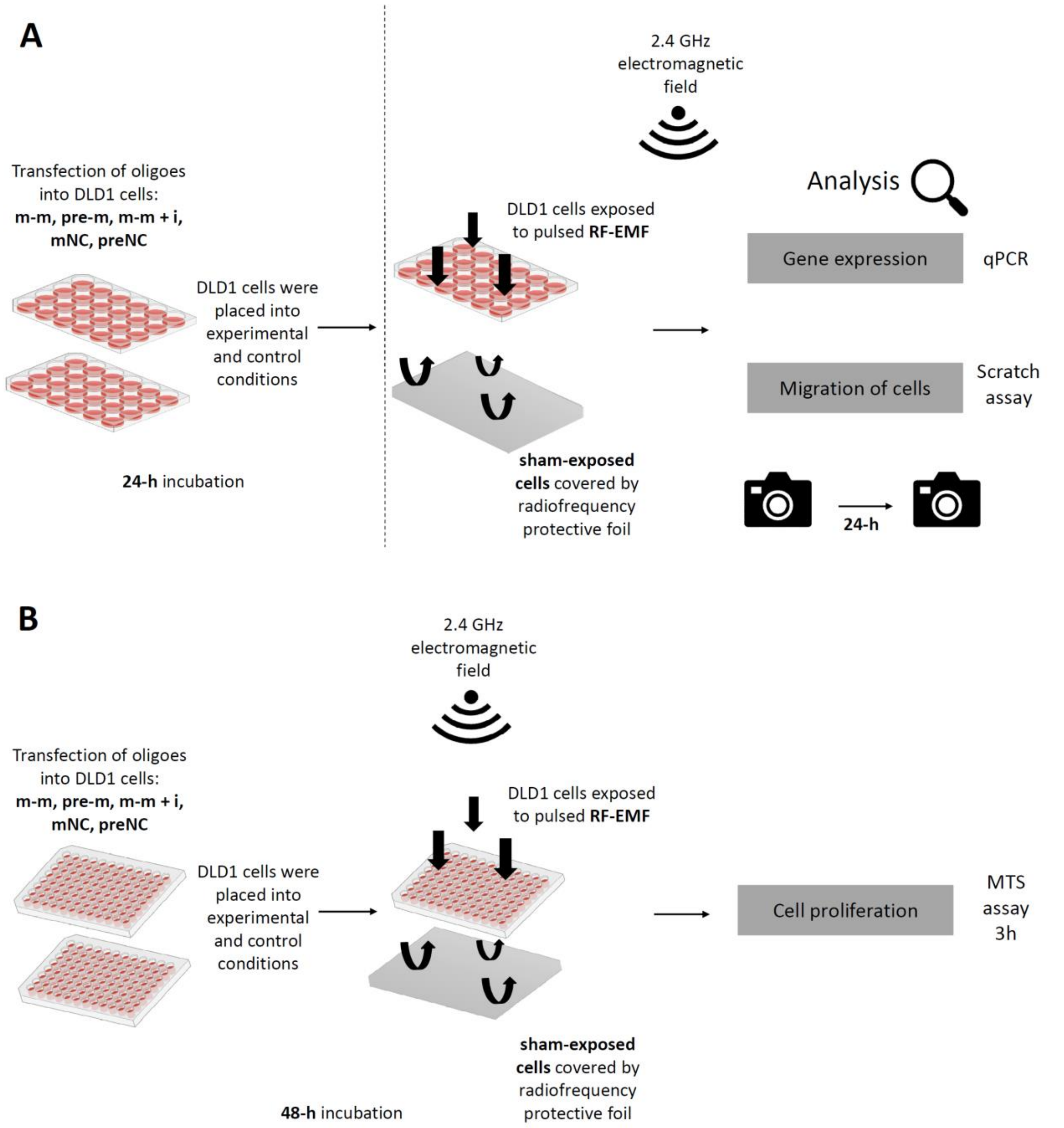

Supplementary Figure S1: Scheme of experimental set up of scratch assay followed by gene expression analysis (A) and MTT assay (B). m-m, a mimic of miR-34a mature dominant strand miR-34a-5p; pre-m, a precursor of miR-34a mimic; m-m + i, a miR-34a-inhibitor and m-m; mNC, a negative control of mimic; preNC, a negative control pf precursor; MTS, modified tetrazolium compound assay; RF-EMF, radio-frequency electromagnetic field.
