# Supplementary material for: 2.4 GHz Electromagnetic Field Influences the Response of the Circadian Oscillator in the Colorectal Cancer Cell Line DLD1 to miR-34a-Mediated Regulation
Source: Int J Mol Sci. 2022 Oct 30;23(21):13210. doi: 10.3390/ijms232113210 (PMC9656412; doi:10.3390/ijms232113210)
Supplement: Supplementary file 1 [file ijms-23-13210-s001.zip › Table S1.pdf]

Supplementary Table S1: Primer sequences.

Gene	Strand	Sequence (5′ → 3′)	Accession number	Annealing temperature (°C)
cry1	sense<br>antisense	CCG TCT GTT TGT GAT TCG TG<br>AAG TTA GAG GCG GTT GTC CA	NM_004075.4	49
cry2	sense<br>antisense	GGA GGC TGG TGT GGA AGT AG<br>CGT AGG TCT CGT CGT GGT TC	NM_001127457.2	49
per2	sense<br>antisense	AAT GCC GAT ATG TTT GCG GT<br>GCA TCG CTG AAG GCA TCT CT	NM_022817.1	53
clock	sense<br>antisense	CCG GAA ACA ATA CCT CCA CC<br>CAC ATG AAA CAG ACA CCC CA	NM_001267843.2	53
bmal1	sense<br>antisense	ACT TCC CCT CTA CCT GCT CAA<br>TGT CTT CAT CCA GCC CCA TC	NM_001297724.1	53
sirt1	sense<br>antisense	CCG GAA ACA ATA CCT CCA CC<br>CAC ATG AAA CAG ACA CCC CA	NM_001142498.2	53
survivin	sense<br>antisense	GGA CCA CCG CAT CTC TAC AT<br>GAC AGA AAG GAA AGC GCA AC	NM_001168	53
s17	sense<br>antisense	CAA GAT AGC AGG TTA TGT CAC G<br>AGC TTC AGC ATT TCC TTA GTG T	NM_001021.3	53
miR-34a	sense	GCA GTG GCA GTG TCT TAG	MIMAT0000255	62 (2x)
	antisense	GGT CCA GTT TTT TTT TTT TTT TAC AAC		60 (36x)

cry, cryptochrome; *per2*, period circadian regulator 2; *clock*, circadian locomoter output cycles protein kaput; *bmal1*, brain and muscle ARNT-like 1; *sirt1*, sirtuin 1; *survivin*, baculoviral IAP repeat containing 5; *s17*, ribosomal protein S17; number in parenthesis refers to number of cycles under given temperature.
